# Correction of Diastasis Rectus Abdominis with Tacking the Rectus Sheath and Resection of Excess Skin for Cosmesis

**DOI:** 10.1155/2020/7635801

**Published:** 2020-06-16

**Authors:** Kento Takaya, Noriko Aramaki-Hattori, Hanayo Yabuki, Norihito Wada, Shigeki Sakai, Keisuke Okabe, Kazuo Kishi

**Affiliations:** ^1^Department of Plastic and Reconstructive Surgery, Keio University School of Medicine, Tokyo, Japan; ^2^Department of Surgery, Keio University School of Medicine, Tokyo, Japan

## Abstract

**Introduction:**

We report a case of diastasis rectus abdominis (DRA), in which the improvement of the appearance was obtained by performing extra skin resection. *Case Report*. A 30-year-old woman presented persistent abdominal bulging after her second delivery. She was diagnosed as DRA by computed tomography. We underwent a surgery that tacking the anterior layer of the rectus sheath and resecting excess skin.

**Results:**

There has been no clinical evidence of recurrence, and the patient satisfies her abdominal appearance.

**Conclusion:**

Because DRA is not a true hernia, surgery for DRA should be performed in understanding how patients want to improve their aesthetic appearance.

## 1. Introduction

Diastasis rectus abdominis (DRA) is a state in which the abdominal wall is stretched due to an increase in abdominal pressure, such as that occurs during pregnancy or with obesity, resulting in abdominal bulging [1]. Because DRA is not a true hernia and is not associated with strangulation, it does not necessarily require surgical repair, and there are no defined indications for surgery [2].

## 2. Case Presentation

A 30-year-old woman noted an increase in weight of 20 kg during her second pregnancy, which was delivered by cesarean. Her postpartum weight decreased by 17 kg, but she noted persistent abdominal bulging. Computed tomography showed a separation of the rectus abdominis at the site of the abdominal wall scar. The patient presented to us desiring cosmetic improvement. Her history was significant for a 20 kg weight gain with her first pregnancy, but she had no subsequent abdominal distension. Her medical history was otherwise unremarkable.

A 5 cm vertical surgical scar was noted to extend distally from the umbilicus. The abdomen bulged outward from the level of the lower sternum to below the umbilical fossa (Figure 1), and a 15 cm separation of rectus abdominis was palpated both above and below the umbilical fossa. Computed tomography showed the midline separation of the rectus abdominis muscle around the navel. The rectus abdominis fascia was maintained, and there was no extrusion of abdominal contents. The inter-recti distance (IRD) was 36 mm at 3 cm above the superior border of the umbilicus, 40 mm at the center of the umbilicus, and 36 mm at 2 cm below the inferior border of the umbilicus (Figure 2).

Surgery was performed under general anesthesia. An abdominal midline incision was made on the cesarean scar, and the linea alba was noted to be thinned and being pulled to the left and right sides of the abdomen. The rectus abdominis muscle was separated horizontally. The anterior rectus sheath was sutured in the midline using nonabsorbable suture without the performance of the midline incision on the anterior sheath, and a high-density polyethylene mesh was applied to the linea alba and rectus abdominis muscle. Attention was then turned to the excess skin and subcutaneous adipose tissue, including the pigmented portion of skin. A spindle-shaped incision was used to excise this tissue, with the spindle measuring 15 cm in the horizontal direction (Figure 3).

A drain was placed over the mesh. The superficial fascia was sutured, and skin closure was performed, using size zero polydioxanone suture. Adhesive skin closure strips were applied to the incision. The patient's operative course was uneventful. Computed tomography performed 2 months after surgery showed no recurrence of the swelling of her abdomen. The patient is satisfied with her abdominal appearance (Figure 4). There has been no clinical evidence of recurrence and no complications for one year after surgery.

## 3. Discussion

Surgical repair for DRA is widely performed in Europe and the United States for cosmetic purposes, but it is a little-known clinical condition in Japan [3]. Both laparotomy and laparoscopic methods are used, and various techniques, such as the use of mesh and layered suturing, are employed [4]. Open surgery is often performed by suturing the anterior or posterior sheath of the rectus abdominis muscle with both an absorbable and nonabsorbable suture to form a fistula. Indwelling mesh may be placed at the surgeon's discretion [5]. In laparoscopic surgery, as in the open technique, the posterior surface of the linea alba is sutured to form pleats; mesh is placed in almost all patients. A report comparing open with laparoscopic surgery notes that the latter results in smaller surgical scars does not require placement of an indwelling drain and reduces perioperative complications; however, this report does not have a high level of evidence.

When planning surgery, attention is focused on repairing the fascia, since the cause of the problem is stretching and thinning of the anterior layer of the rectus sheath and the linea alba. Subcutaneous liposuction is sometimes combined with this repair [6]. In our patient, we repaired the abdominal wall, and then, we tightened the entire abdominal area by removing excess skin and subcutaneous fat, allowing for an aesthetically pleasing result.

Reported complications of abdominoplasty include hematoma and seroma formation, wound infection, skin flap necrosis, and hypertrophic scarring [7]. Generally, the recurrence rate is low and complications are rare, but any surgery requires a sufficient explanation of the risks and benefits to the patient [8].

Additionally, our method has a limitation. It is a long vertical scar that remains in the midline of the abdomen. In this case, a scar after the caesarean section was originally found under the umbilicus, and a remarkable skin laxity was observed from the upper part of the umbilicus to the lower abdomen, so a long scar was needed to remove it. However, new scarring from the surgical procedure should be minimized for further cosmetically superior results in future cases. Laparoscopic surgery is an alternative to minimize postsurgical scarring, but at the risk of postoperative complication such as organ damage and adhesive ileus. If skin is less lax, we may need to consider a previously reported approach to the rectus anterior sheath through a small incision around the navel [9, 10].

We repaired the abdominal wall in this patient with DRA by tacking the anterior layer of the rectus sheath and by placing mesh. We also resected the excess skin to improve cosmesis and achieve a high level of patient satisfaction. We think that it is necessary to consider the possibility of DRA when planning surgery for a patient with abdominal bulging extending beyond an operative scar.

## Figures and Tables

**Figure 1:**
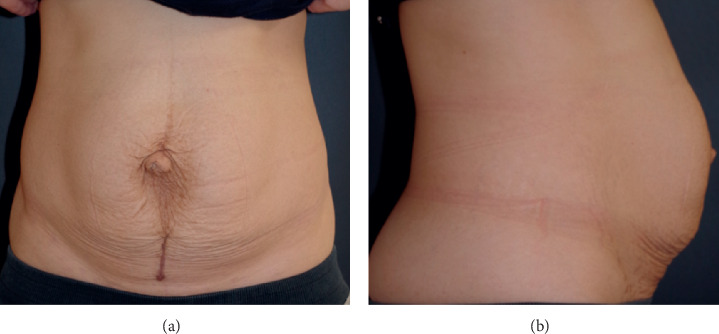
A 5 cm vertical surgical scar is present distal to the umbilicus. Significant midline abdominal bulging is noted to extend beyond the level of the scar.

**Figure 2:**
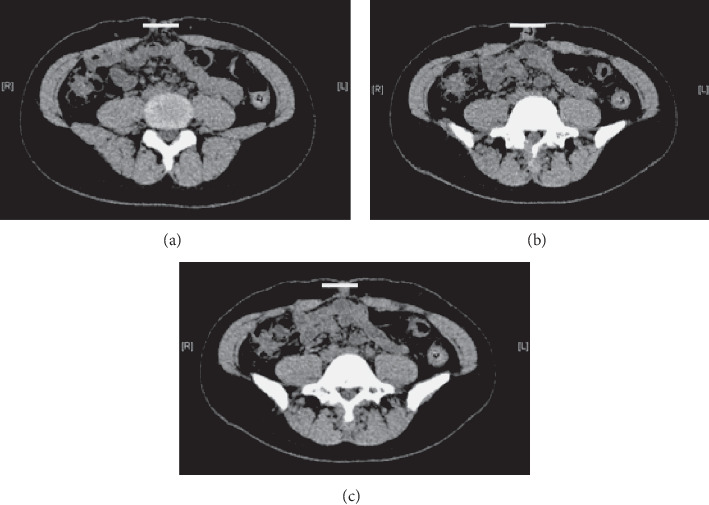
Preoperative abdominal computed tomography: (a) 3 cm above the superior border of the umbilicus; (b) center of the umbilicus; (c) 2 cm below the inferior border of the umbilicus. The inter-rectus distance is indicated by a white line.

**Figure 3:**
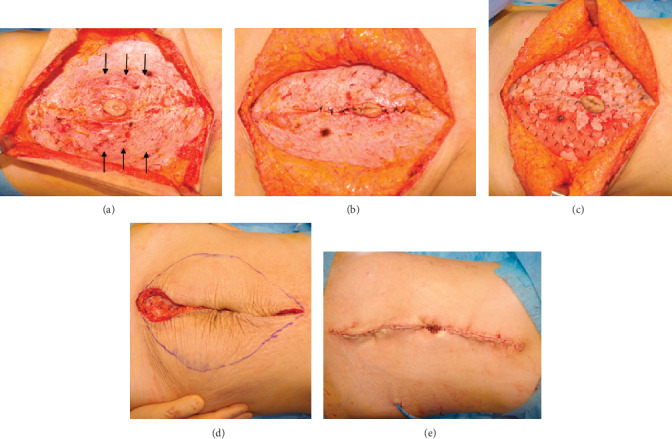
Intraoperative photographs. (a) The rectus abdominis muscle is displaced to the left and right. The peritoneum is intact. (b) The anterior rectus muscle is sutured. (c) A mesh is applied to the rectus abdominal muscle. (d) Excess skin is removed in a spindle shape. (e) Closure of the incision.

**Figure 4:**
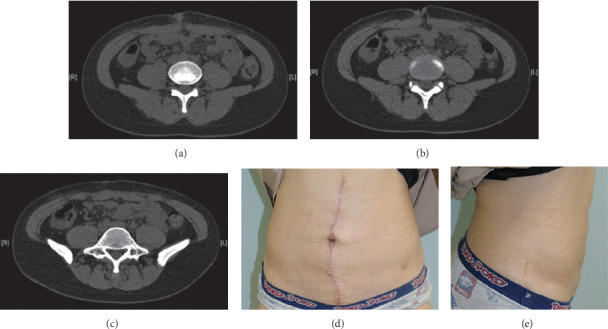
(a–c) Postoperative abdominal computed tomography. (a) 3 cm above the superior border of the umbilicus; (b) center of the umbilicus; (c) 2 cm below the inferior border of the umbilicus. There is no apparent recurrence of the hernia. (d-e) Appearance three months after surgery. The patient's abdominal bulge has resolved.

## Data Availability

Data sharing not applicable to this article as no datasets were generated or analyzed during the current study.
